# DNA Methylation Changes Separate Allergic Patients from Healthy Controls and May Reflect Altered CD4^+^ T-Cell Population Structure

**DOI:** 10.1371/journal.pgen.1004059

**Published:** 2014-01-02

**Authors:** Colm E. Nestor, Fredrik Barrenäs, Hui Wang, Antonio Lentini, Huan Zhang, Sören Bruhn, Rebecka Jörnsten, Michael A. Langston, Gary Rogers, Mika Gustafsson, Mikael Benson

**Affiliations:** 1 The Centre for Individualized Medicine, Linköping University Hospital, Linköping University, Linköping, Sweden; 2 Department of Pediatrics, Sahlgrenska Academy, University of Gothenburg, Gothenburg, Sweden; 3 Mathematical Sciences, Chalmers University of Technology, University of Gothenburg, Gothenburg, Sweden; 4 Department of Electrical Engineering and Computer Science, University of Tennessee, Knoxville, Tennessee, United States of America; 5 National Institute for Computational Sciences, University of Tennessee, Knoxville, Tennessee, United States of America; HudsonAlpha Institute for Biotechnology, United States of America

## Abstract

Altered DNA methylation patterns in CD4^+^ T-cells indicate the importance of epigenetic mechanisms in inflammatory diseases. However, the identification of these alterations is complicated by the heterogeneity of most inflammatory diseases. Seasonal allergic rhinitis (SAR) is an optimal disease model for the study of DNA methylation because of its well-defined phenotype and etiology. We generated genome-wide DNA methylation (*N_patients_* = 8, *N_controls_* = 8) and gene expression (*N_patients_* = 9, *N_controls_* = 10) profiles of CD4^+^ T-cells from SAR patients and healthy controls using Illumina's HumanMethylation450 and HT-12 microarrays, respectively. DNA methylation profiles clearly and robustly distinguished SAR patients from controls, during and outside the pollen season. In agreement with previously published studies, gene expression profiles of the same samples failed to separate patients and controls. Separation by methylation (*N_patients_* = 12, *N_controls_* = 12), but not by gene expression (*N_patients_* = 21, *N_controls_* = 21) was also observed in an *in vitro* model system in which purified PBMCs from patients and healthy controls were challenged with allergen. We observed changes in the proportions of memory T-cell populations between patients (*N_patients_* = 35) and controls (*N_controls_* = 12), which could explain the observed difference in DNA methylation. Our data highlight the potential of epigenomics in the stratification of immune disease and represents the first successful molecular classification of SAR using CD4^+^ T cells.

## Author Summary

T-cells, a type of white blood cell, are an important part of the immune-system in humans. T-cells allow us to adapt our immune-response to the various infectious agents we encounter during life. However, T-cells can also cause disease when they target the body's own cells, *e.g.* Psoriasis, or when they react to a harmless particle or ‘antigen’, *i.e.* allergy. Much evidence supports an environmental, or ‘epigenetic’, component to allergy. Surprisingly, although allergy is viewed as a T-cell disease with an epigenetic component, no studies have identified epigenetic differences between healthy individuals and allergic individuals. Using a state-of-the-art genome-wide approach, we found that we could clearly and robustly separate allergic patients from healthy controls. It is often assumed that these changes reflect changes in DNA methylation in a given type of cell; however such differences can also result from different mixtures of T-cell subtypes in the samples. Indeed, we found that allergic patients had different proportions of T-cell sub-types compared to healthy controls. These changes in T-cell proportions may explain the difference in DNA methylation profile we observed between patients and controls. Our study is the first successful molecular classification of allergy using CD4^+^ T cells.

## Introduction

The modest effects of genetic variants in inflammatory diseases indicate the importance of epigenetic mechanisms like DNA methylation to disease pathology. However, studies of inflammatory diseases have shown conflicting results. In monozygotic twins discordant for multiple sclerosis (MS), no significant differences in DNA methylation profile were found [1]. A more recent study of monozygotic twins discordant for psoriasis identified widespread differences between siblings [2]. Other studies of autoimmune diseases have reported varying findings [3]. Discordant monozygotic twin studies benefit from a constant genetic background on which to identify disease-associated epigenetic changes. However, intrinsically, such studies tend to involve small samples sizes, and may thus lack the power to detect small, rare, disease-associated changes in DNA methylation. The variation between studies may be due to disease heterogeneity, variations in disease course and the confounding effects of treatment, and as the disease causing agent is unknown, it is difficult to experimentally model disease pathogenesis.

By contrast, seasonal allergic rhinitis (SAR) occurs at defined time points each year and the disease causing agent, pollen, is known. These unique features of SAR permit analysis of CD4^+^ T-cells from SAR patients during and after the pollen season *in vivo*, and by allergen-challenge *in vitro* [4]. An epigenetic component in SAR is supported by its increasing prevalence in the developing world, failure of genome-wide association studies to identify a consistent genetic component to the disease, and frequent discordance for SAR between monozygotic twins [5], [6]. DNA methylation changes at numerous loci are required for appropriate differentiation of naïve CD4^+^ T-cells into CD4^+^ T effector cell subtypes [7].

We generated genome-wide expression (*N_patients_* = 9, *N_controls_* = 10) and DNA methylation (*N_patients_* = 8, *N_controls_* = 8) profiles for CD4^+^ T-cells from untreated SAR patients and healthy controls, both during and outside the pollen season. Consistent with previous studies, we found that CD4^+^ T-cell gene expression profiles were poor classifiers of SAR. However, we observed clear and robust separation of patients and controls by DNA methylation signature, both during and outside the pollen season. Separation by methylation (*N_patients_* = 12, *N_controls_* = 12), but not by gene expression (*N_patients_* = 21, *N_controls_* = 21) was also observed in an *in vitro* model system in which purified PBMCs were challenged with allergen in culture. Moreover, we found that these methylation profiles were significantly associated with disease severity in patients during season, and may be due to differing proportions of central memory CD4^+^ T-cells (*N_patients_* = 35, *N_controls_* = 12). This, to our knowledge, is the first successful molecular classification of SAR using CD4^+^ T-cells, and highlights the potential of genome-wide epigenetic technologies in the stratification of immune disease.

## Results and Discussion

### The DNA methylation profile of CD4^+^ T-cells separates allergic patients from healthy controls following allergen-challenge *in vitro*

**Figure 1:**
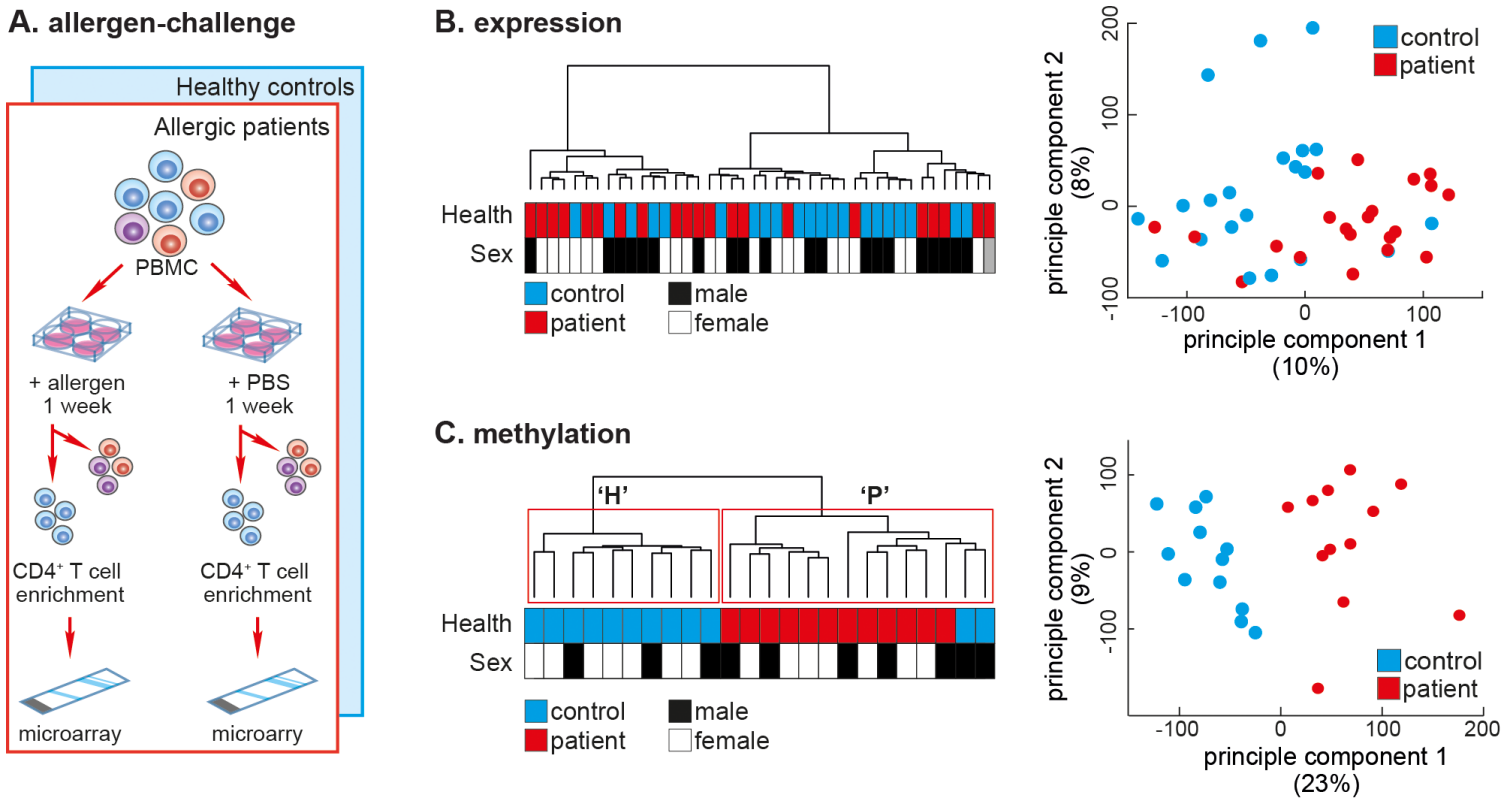
The DNA methylation profile of allergen-challenged CD4^+^ T-cells separates SAR patients from healthy controls. (**A**) Allergen-challenge assay. Peripheral blood mononuclear cells were isolated from healthy individuals and SAR patients and challenged *in vitro* with either allergen (pollen) or diluent (PBS). One-week post-challenge total CD4^+^ T-cells were isolated by MACS negative cell sorting. Genomic DNA and total RNA were isolated from the purified cells and cDNA or bisulfite-converted DNA was applied to gene expression or DNA methylation arrays, respectively. (**B**) Unsupervised hierarchical clustering of gene expression data of CD4^+^ T-cells isolated after allergen-challenge of PBMCs from SAR patients (*N* = 21) and healthy control subjects (*N* = 21) collected outside the pollen season (left panel).. Sample annotation is illustrated by colored boxes below the dendrogram. Principle components analysis of the same gene expression data fails to cluster data by disease status (right panel). (**C**) Unsupervised hierarchical clustering of quantitative genome-wide DNA methylation data of CD4^+^ T-cell DNA isolated after allergen-challenge of PBMCs from SAR patients (*N* = 12) and healthy control subjects (*N* = 12) collected outside the pollen season (left panel). Repeated bootstrap resampling of the data to calculate *P*-values for each cluster revealed that the two main clusters (H & P) were significantly supported by the data (*P*<0.05). Principle components analysis of the same DNA methylation data also revealed clear separation by disease state along the main principle component (right panel).

Seasonal allergic rhinitis (SAR) is a powerful disease model because, (1) SAR's clear clinical manifestations make it easy to assess disease severity, (2) the affected cell type, CD4^+^ T-cells, can be obtained from patients when they are symptom-free (outside the pollen season) and compared to the same cell type in the same individual when symptomatic (during the pollen season). We aimed to test the ability of CD4^+^ T-cell DNA methylation to separate SAR patients from healthy controls. The cohorts used in the study are outlined in Supplemental Figure S1. In a previous study, we obtained PBMCs from adult SAR patients (*N* = 21; age = 25.4 years±7.8 SD) and healthy controls (*N* = 21; age = 25.7 years±10.1 SD) outside the pollen season, and challenged these cells with either grass pollen extract or diluent (PBS) (Figure 1A). Seven days post-challenge, total CD4^+^ T-cells were isolated by magnetic-activated cell sorting (MACS, positive-selection), and the mRNA expression profile determined by gene expression microarray (GEO: GSE50223). Consistent with several previous studies, we were unable to separate patients and controls after challenge with allergen by CD4^+^ T-cell mRNA expression profile [6], [8], [9] (Figure 1B).

Here, PBMCs from a new cohort of SAR patients (*N* = 12; age = 28.3 years±12.1 SD) and healthy controls (*N* = 12; age = 27.3 years±10.7 SD) were allergen-challenged, after which purified CD4^+^ T-cell DNA was analysed using Illumina HumanMethylation27 DNA methylation microarrays. Unsupervised hierarchical clustering of samples by genome-wide DNA methylation profiles resulted in two groups, ‘H’ (healthy controls) and ‘P’ (patients), clearly separating samples by disease-state (Figure 1C, left panel). Consensus clustering, whereby the data are repeatedly re-sampled and re-clustered, found that clusters ‘H’ and ‘P’ were reproducibly and stably identified (*P*<0.05). We confirmed this separation in the data using principal component analysis (PCA), which also revealed a clear separation between allergen-challenged CD4^+^ T-cells from patients versus controls (Figure 1C, right panel). A leave-one out (LOO) cross-validation found that methylation data accurately classified all samples as patient or healthy control (χ^2^;*P*<0.0001). DNA methylation and gene expression signatures did not separate patients and healthy controls after diluent challenge (Figure S2).

### The DNA methylation profile of *in vivo* CD4^+^ T-cells separates allergic patients from healthy controls both during and outside the pollen season

Though striking, the results obtained from *in vitro* allergen-challenge of PBMCs may be confounded by cell culture effects. To verify the observations made after *in vitro* allergen-challenge, the mRNA expression and DNA methylation profiles of *in vivo* CD4^+^ T-cells were determined in a new cohort of SAR patients and healthy controls (GEO: GSE50387). In this experiment we used Illumina HumanMethylation450k methylation arrays, which quantitatively assess 450,000 CpG sites across the genome, with over half the probes targeting CpGs outside gene promoters and a quarter of all probes targeting CpGs in non-genic regions. CD4^+^ T-cells were purified from fresh blood collected from SAR patients and healthy controls by negative magnetic cell sorting both during and outside the pollen season in the same calendar year. Symptom scores for patients were recorded at the time of collection (Table S1). DNA and total RNA were harvested simultaneously from each sample. Subsequently, cDNA and bisulfite converted DNA was applied to Illumina HT12 expression microarrays (*N_patients_* = 9, *N_controls_* = 10; both during and outside the pollen season) and HumanMethylation450k microarrays microarrays (*N_patients_* = 8, *N_controls_* = 8; both during and outside the pollen season), respectively.

**Figure 2:**
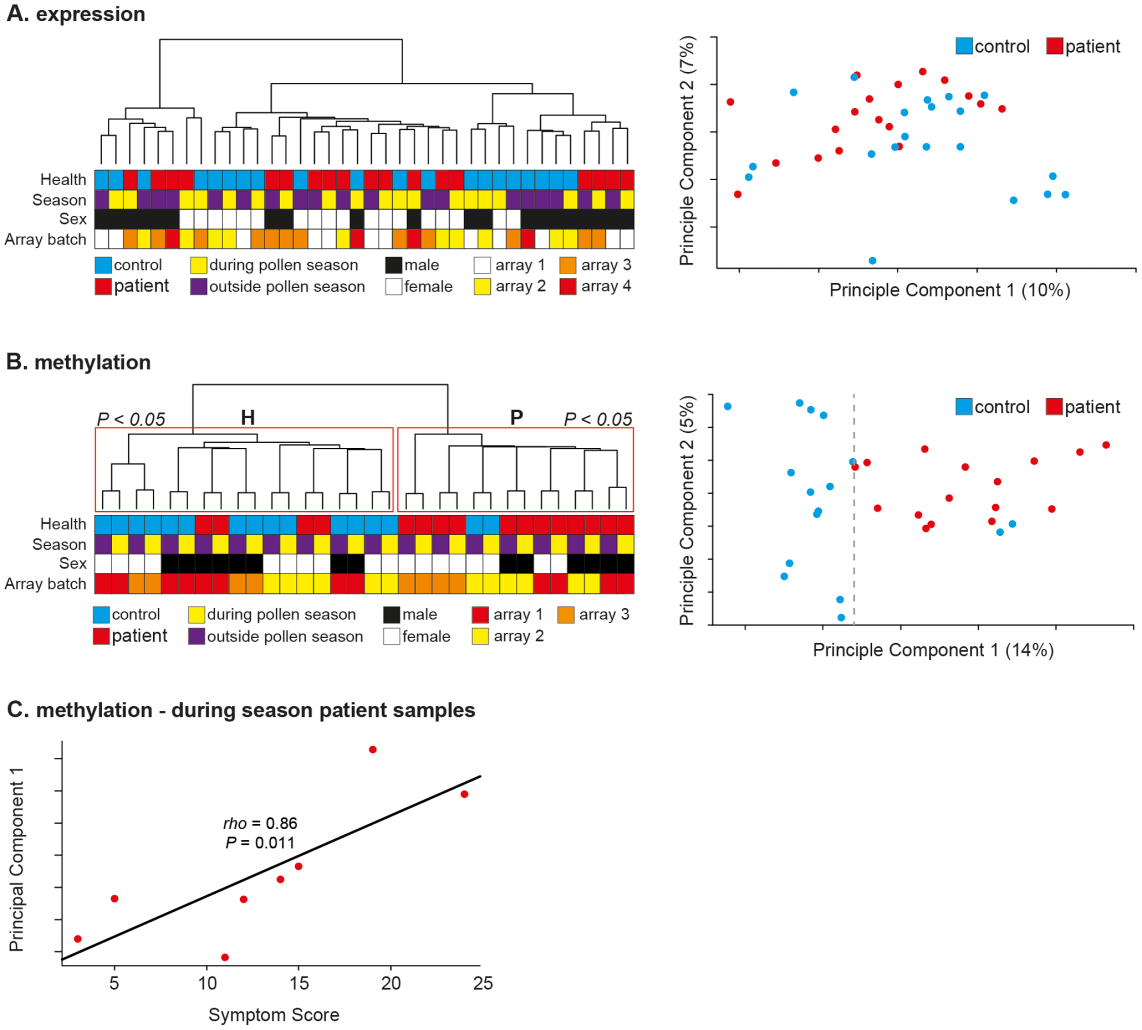
*In vivo* CD4^+^ T-cell methylation separates patients from controls during and outside the pollen season. (**A**) Unsupervised hierarchical clustering of gene expression data of CD4^+^ T-cells from SAR patients (*N* = 9) and healthy control subjects (*N* = 10) both during and outside the pollen season (left panel). Sample annotation is illustrated by colored boxes below the dendrogram. Principle components analysis of the same gene expression data failed to cluster samples by disease status (right panel). Array batch indicates which samples were analyzed on the same microarray slide. (**B**) Unsupervised hierarchical clustering of quantitative genome-wide DNA methylation data of CD4^+^ T-cells from SAR patients (*N* = 8) and healthy control subjects (*N* = 8) both during and outside the pollen season (left panel). Repeated bootstrap resampling of the data to calculate *p*-values for each cluster revealed that the two main clusters (H & P) were significantly supported by the data (*P*<0.05). Principle components analysis of the same DNA methylation data also revealed clear separation by disease state along the main principle component (right panel). Array batch indicates which samples were analyzed on the same microarray slide. (**C**) Plot of patient symptom score during season with PCA1 value revealed a highly significant correlation (Spearman's *rho* = 0.86, *P* = 0.01).

Unsupervised hierarchical clustering of samples by mRNA expression profile did not result in separation of samples by disease state either during or outside the pollen season (Figure 2A, left panel). No effect of array batch or sex was evident in the observed clusters (Figure 2A, left panel). These findings are in line with those reported here (Figure 1B) and in numerous previous studies of CD4^+^ T-cells in allergy and other autoimmune diseases [6], [8], [9]. PCA of the gene expression data also failed to distinguish between SAR patients and healthy controls (Figure 2A, right panel). Differentially expressed genes were identified as those showing differential expression between patients and controls (Mann-Whitney *U*-test; *P*<0.05, unadjusted) both during and outside the pollen season (357 genes identified). The relative changes observed across all genes were small (mean absolute fold change = 0.39, SEM ± 0.01). Gene Ontology (GO) term analysis failed to identify any significantly enriched gene annotation clusters after correction for multiple correction. However, four of the top 10 annotations related to lymphocyte activation (Figure S3A). All four clusters consisted of mixtures of the same seven genes, namely; *HSPD1*, *TGFBR2*, *CD134* (*TNFRSF4, OX40*), *CD45* (*PTPRC*), *SPN* (*CD43*), *IL2RA*, *IL13RA1*. This group of genes is highly relevant; *CD43* ^−/−^ mice show a pronounced Th2 phenotype and exhibit increased in inflammation in two allergic mouse models [10] and association studies have identified both *IL2RA* and *TGFBR2* as susceptibility loci for allergy and asthma [11]. *CD143* is a regulator of CD4^+^ T-cell memory and *CD45* isoforms are key markers of memory CD4^+^ T-cells, though it is not possible to determine the exact isoform mis-expressed due to the positioning of the gene expression probe in the 5′ UTR of *CD45* [12]. To determine the relationship between the observed changes in gene expression and DNA methylation we determined the average promoter methylation for all genes represented on the 450K methylation array. Changes in gene expression and associated promoter DNA methylation between patients and controls were not significantly negatively correlated (Pearson's *r* = 0.14, *P* = 0.07), as would be expected if DNA methylation were acting to silence transcription (Figure S3B).Thus, although it was not possible to separate patients from controls by gene expression profile, a small set-of disease-relevant miss-expressed genes were identified.

In contrast, unsupervised hierarchical clustering of all probes by DNA methylation profile resulted in clear separation of the samples by disease state, regardless of season (during or outside pollen season); seven of eight healthy controls grouped in cluster ‘H’, and six of eight patient samples grouped in cluster ‘P’ (Figure 2B, left panel). Consensus clustering identified clusters ‘H’ and ‘P’ with high statistical confidence (Multiscale Bootstrap Resampling; *P*<0.05). Interestingly, the paired measurements (during and outside season) of each sample also clustered neatly together; again implicating disease status as the major modifier of DNA methylation profile between patients and controls samples, while also highlighting the reproducibility and accuracy of the data (paired samples were collected and processed at least five months apart) (Figure 2, left panel). PCA resulted in even clearer separation of samples along the main principle component, with only one healthy sample clustering among the patient samples (Figure 2, right panel). LOO analysis correctly classified samples as patient or healthy control in all samples collected outside season (χ^2^;*P*<0.0001), and all but two healthy samples collected during season (χ^2^;*P* = 0.01). PCA1 explained approximately 14% of the variation in the data and 3-times the variation explained by PCA2 (5%). Although our findings indicated that disease status was the major mediator of DNA methylation differences between patients and healthy controls an effect of pollen season was also evident. With the exception of one outlier, healthy samples clustered tightly together along PCA1, whereas patient samples showed greater spread along PCA1 (Figure 2B, right panel). Given this observation, we tested if the position along PCA1 of patient samples was associated with patient symptom score during season. Symptom scores for each patient are listed in Supplemental Table S1. Significantly, we found that symptom score explained 74% of the variation in patient sample variation along PCA1, a strong and highly significant correlation (Spearman's *rho* = 0.86, *P* = 0.011) (Figure 2C). To our knowledge, such a strong association between individual or genome-scale markers and symptoms has not been previously been described in allergy, nor in other inflammatory diseases. However, given the small sample size (*N_patients_* = 8) of the study presented here, the use of DNA methylation as a marker of disease severity needs to be tested in a much larger cohort. The association with disease severity may also explain the difficulty in identifying an epigenetic component to other immune-related diseases in which the disease course is more variable and complex to assess. However, as patient symptoms can vary dramatically during the pollen season it is important that the observed correlation between DNA methylation and symptom severity is tested at several time-points during a pollen season to validate the robustness of the preliminary observation reported here. If our findings are applicable to other inflammatory diseases, an important implication is that DNA methylation may help to stratify such diseases.

The observed differences in DNA methylation between patients and controls were small (mean absolute change = 1.2%±2.3 SD), bi-directional, and genome-wide, 12,000 probes (3.5% of all probes) were found to have changed significantly (Mann-Whitney U-test; *P*<0.01; unadjusted) (Figure S4A). Indeed, the 1,000 most significantly altered probes changed by only ±10% (Figure S4B). Given the small size of the observed methylation differences and the known technical variation associated with 450K methylation arrays [13], we selected five CpG loci for validation by pyrosequencing in 4 SAR patients and 4 healthy controls. We selected three CpGs that showed significant methylation differences between patients and healthy controls (among top 50 altered probes) which were also located in annotated gene promoters (*PIEZO1* promoter CpG, *RPP21* promoter CpG, *HLA-DMA* promoter CpG) and 2 control CpG loci (unmethylated CpG; *GAPDH* promoter & methylated CpG; *CD74* promoter). Pyrosequencing primers are listed in Table S2. Array methylation was highly significantly correlated with pyrosequencing methylation for all CpG loci (Spearman's *rho* = 0.98, *P*<10^−6^) (Figure S4C), and the absolute difference in methylation measurements between the array and pyrosequencing across all CpGs was small (median difference = 2.41%±3.2 MAD; mean difference = 4.1%±4 SD). The methylated (Figure S4D) and unmethylated (Figure S4E) control CpGs were also validated by pyrosequencing, although agreement between array and pyrosequencing was slightly better for the unmethylated CpG site. Critically, pyrosequencing confirmed the direction and scale of DNA methylation change observed between patients and controls by 450K methylation array at the three disease-associated test loci (Figure S4F–S4H).

Our findings agree with a recent quantitative study of genome-wide methylation in CD4^+^ T-cells from monozygotic twins discordant for the autoimmune disease, psoriasis, in which affected and unaffected siblings were distinguished by numerous, small, bi-directional changes in DNA methylation, with no one CpG exhibiting a significant change in DNA methylation level [2]. Moreover, and very recent study of DNA methylation in CD4^+^ T-cells from patients with the autoimmune disease Systemic Lupus Erythematosus (SLE) also reported widespread small changes in DNA methylation [14]. Interestingly, we found that the genic location of significantly altered probes was enriched for gene-bodies and non-genic regions (χ^2^; *P*<0.0001) (Figure S5A). This highlights the importance of using unbiased genomic technologies; as assays targeted towards detection of large changes in DNA methylation in promoter regions may miss subtle but informative changes in other genomic compartments. To further dissect the genomic compartmentalization of the observed changes in DNA methylation we focused on regulatory elements whose function is known to be modified by DNA methylation, namely promoters, DNaseI hypersensitive sites (DHS) and enhancer elements. Interestingly, differentially methylated probes appeared enriched in annotated enhancers compared to those located in DHSs and promoters (χ^2^; P<10^−5^) (Figure S5B). This enrichment was observed both during and outside the pollen season (Figure S5B). Enhancer probes differentially methylated during and outside the pollen season showed a significant overlap (Fisher's exact test; *P*<0.001) (Figure S5C). Significantly, those enhancer probes differentially methylated both during and outside the pollen season (*N* = 960) showed a clear tendency towards loss of methylation in patients in contrast to the bi-directional changes observed for all differentially methylated probes (Figure S5D). As the activity of many enhancer elements and consequently their associated genes, is affected by DNA methylation, this finding may reflect the epigenetic re-modeling of enhancer elements in CD4^+^ T-cells. However, as most enhancers are only active in a small number of tissues we cross-referenced the observed differentially methylated enhancers with a recently published experimentally determined set of Th1-, Th2- and Th0-specific enhancers [15], [16]. Only 1 of the differentially methylated enhancer probes (*N* = 960) mapped to the Th1/Th2/Th0 enhancers. Thus, is seems unlikely that the observed changes in enhancer methylation have function gene expression consequences in Th2 cells, the key pathogenic cell-type in allergy. However, as Th2 cells constitute <3% of the total CD4^+^ T-cell population, we cannot exclude large functionally relevant changes in DNA methylation at enhancers in other CD4+ T-cell subsets.

**Figure 3:**
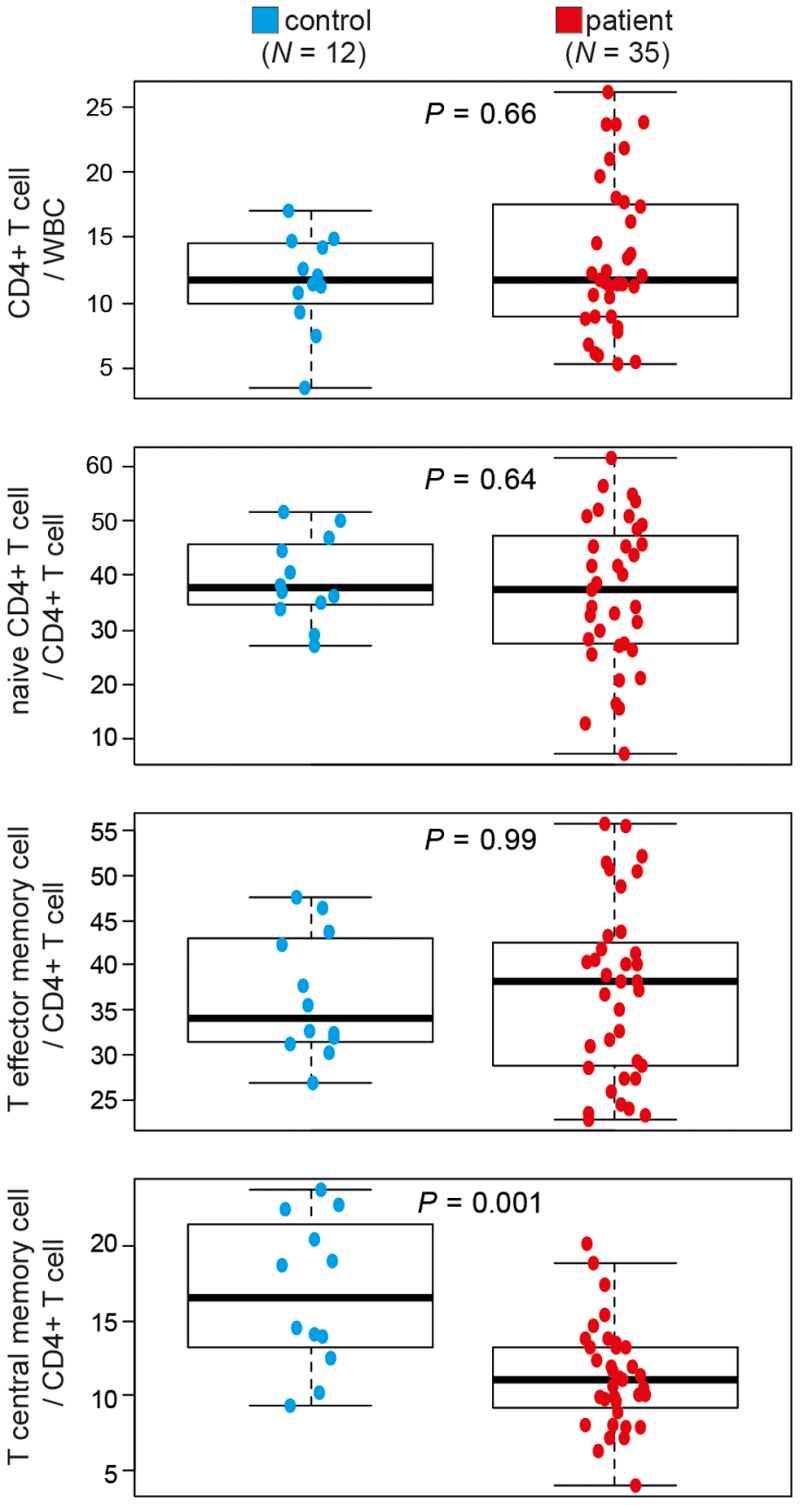
CD4^+^ T-cell subsets differ between healthy controls and patients with seasonal allergic rhinitis. Total CD4^+^ T-cells, naïve CD4^+^ T-cells, CD4^+^ T central memory cells and T effector memory cell numbers in patients with seasonal allergic rhinitis (SAR) (*N* = 35) and healthy controls (*N* = 12) were determined by flow cytometry. WBC, white blood cells. Differences between samples were determined by Mann-Whitney U-test.

Differences in DNA methylation are often assumed to reflect changes in DNA methylation in a given type of cell; however such differences can also result from changes in the proportions of cell types in samples. Indeed, a recent study reported that a significant proportion of the DNA methylation differences observed between breast tumours (*N* = 248) reflected the number of infiltrating T-cells, and was significantly associated with prognosis [17]. Thus, the observed, small, genome-wide and bi-directional changes in DNA methylation between SAR patients and controls may reflect changes in the proportions of CD4^+^ T-cell sub-populations between patients and controls. Given that DNA methylation profile clearly separates samples by disease-state both during and outside the pollen season we hypothesized that the observed differences may have been due to differences in memory CD4^+^ T-cell subsets. Indeed, we have previously observed a reduction in CD4^+^ central memory T-cells (T_CM_) in a small cohort of allergic patients [18]. Here we extended this study, using FACS analysis to quantify CD4^+^ T-cell subsets in an additional 26 subjects (*N_patients_* = 35, *N_controls_* = 12) (Figure S6). SAR patients were significantly depleted for CD4^+^ central memory T-cells (T_CM_) (11% of total CD4^+^ T-cells) relative to healthy individuals (18.7% of total CD4^+^ T cells), a reduction of 41% in the T_CM_ population in patients relative to controls (Mann-Whitney; *P* = 0.001) (Figure 3). The values for T_CM_ cells in healthy individuals reported here are similar to those reported in independent studies [19], affirming the accuracy of our methodology. Altered T_CM_ proportions are being implicated in a growing number of immune-related diseases [19]–[21]. This finding was also consistent with enrichment for the Gene Ontology (GO) term, ‘negative regulation of memory T-cell differentiation’, found when genes containing the 100 most significantly changed probes were analyzed (Figure S5E, right panel). However, given the enrichment for altered probes in non-genic and non-promoter regions of genes, the results of such GO term analyses must be viewed with caution. As the quantification of CD4^+^ T-cell subsets and DNA methylation profiling were performed on different cohorts of patients and controls, conclusions drawn from these independent observations must be cautious. Determination of DNA methylation profile, CD4^+^ T-cell subset structure and symptom severity in a large cohort of patients and controls at multiple time-points during and outside the pollen season would allow a more robust analysis of the inter-dependence of these variables. Although our results suggest that differences in T-cell sub-populations may contribute to the observed differences in DNA methylation between SAR patients and controls, they do not exclude a direct role for DNA methylation changes within CD4^+^ T-cell subsets. Analysis of gene expression and DNA methylation in each memory CD4^+^ T-cell subset in a large cohort of patients and healthy controls is required to directly test the association between CD4^+^ T-cell subset changes and observed changes in total CD4^+^ T-cell methylation. It is technically challenging to obtain sufficient numbers of each subtype of CD4^+^ T-cell from standard (40 mL) blood samples. An alternate future strategy may be to determine reference genomes for each CD4^+^ T-cell subtype and use these subtype specific methylomes to estimate sub-type proportions from a total CD4^+^ T-cell methylome [22]. This approach has recently been successfully applied to the study of rheumatoid arthritis [23].

The ability to separate patients and healthy controls by quantitative DNA methylation array, but not by gene expression array is interesting. Assuming little or no change in DNA methylation or gene expression profiles within CD4^+^ T-cell subtypes, small changes in the proportions of subtypes should result in very subtle changes in both the DNA methylation and gene expression profiles of total CD4^+^ T-cells. These subtle changes can be detected by the DNA arrays, given **1**) their sensitivity – they are quantitative and accurate, and **2**) their power – they assess 450,000 CpG sites. Gene expression arrays are not quantitative and have a restricted dynamic range of values which is often determined by probe design, not gene transcript levels. Moreover, whereas gene expression arrays typically have probes targeting ∼40,000 transcripts, only a small proportion of these are actually expressed (informative) in any given cell type, further reducing the power of the assay. Moreover, DNA methylation profiles are very stable in normal somatic cells, showing less inter-individual variation than RNA levels. Thus, whereas detecting small changes (<5%) in CD4^+^ T-cell substructure by gene expression microarray is theoretically possible, it would require a very large sample size; much greater than that used in this study (*N_patients_* = 9, *N_controls_* = 10; both during and outside the pollen season). Our results support the use of both DNA methylation arrays and gene expression arrays simultaneously, particularly, where cell-type composition may contribute to the molecular signature of the disease.

Epigenetic regulation plays a key role in Th differentiation. Pronounced changes in DNA methylation patterns are observed at several key loci during helper T cell differentiation [24]. Up-regulation of *Il4*, *IL5*, and *IL13* gene expression in Th2 cells is accompanied by a pronounced loss of DNA methylation and gain of permissive histone marks across the locus [25], [26]. Similarly, the promoter of the Th1 gene, *Ifng*, is unmethylated in Th1 cells, but hypermethylated in Th2 cells, reinforcing helper T-cell identity. DNA demethylation also occurs at regulatory regions of the *FOXP3* gene in Treg cells [27], and at the *IL17A* promoter in Th17 cells [28]. Allergy involves an inappropriate Th2 response to a benign allergen such as pollen [29], and several observations point to a key role for epigenetics in the pathogenesis of SAR. Murine studies have established that a diet rich in methyl donors, such as folic acid enhances allergic airway disease in progeny [30], and knock-out studies of the DNA methyltransferase, Dnmt3a, resulted in dysregulation of important Th2 cytokines, including *Il13*, and increased inflammation in a mouse model of asthma [31]. In humans, large meta-analysis of GWAS identified few loci associated with SAR [32], and those loci identified did not contain genes encoding Th2 genes or other genes of known relevance for SAR. A recent study identified stable and functional DNA demethylation at the key regulatory gene *FOXP3* in patients cured by specific immunotherapy (SIT) [33], directly linking disease reversal with DNA demethylation. Recent studies have reported differences in DNA methylation in airway epithelial cells between asthmatic children and healthy controls [34] and reported that methylation levels at the key cytokine gene, *IL2*, in cord blood was associated with asthma exacerbations in childhood [35]. However, systematic clinical studies of the genome-wide distribution and role of DNA modifications in Th differentiation in SAR are lacking.

Analysis of the transcriptome of CD4^+^ T-cell subsets have failed to identify clear and reproducible differences between patients and healthy controls in several immune-diseases [6], [8], [9]. The circulating total CD4^+^ T-cell population is complex, comprised of several subsets that vary markedly between individuals. This complexity renders identification of subtle allergy-specific transcriptional signals challenging with current approaches. Unlike gene expression microarrays which typically assay 20,000–40,000 transcripts and have a small dynamic range, the DNA methylation microarrays employed here assay ∼450,000 individual CpG sites with quantitative accuracy. We suggest that quantitative DNA methylation microarrays can act as a proxy measure of the cell-population structure of samples, and as such, may be powerful analytical tools for diseases in which the proportions of different cell sub-types is likely to be of pathogenic significance such as immune-diseases and cancer [2], [17], [19]. Indeed, a very recent publication of genome-wide methylation in CD4^+^ T-cells from patients with Systemic Lupus Erythematosus (SLE), identified similar, small widespread changes in DNA methylation and associated these epigenetic changes with changes to CD4+ T-cell populations in SLE patients [14]. This exciting finding is completely consistent with the preliminary results reported here and suggests that alterations in CD4^+^ T-cell populations may be a general feature of many immune diseases. As CD4^+^ T-cell population structure and DNA methylation profiles reported here were determined in different cohorts, simultaneous analysis of the DNA methylation and gene expression profiles of CD4^+^ T-cells in the same cohort of patients and controls is required to directly determine the contribution of changes in subtype structure between patients and controls to the observed difference in total CD4^+^ T-cell methylation. Our findings highlight the potential to stratify immune diseases with DNA methylation.

## Materials and Methods

### Ethics statement

*In vitro* and *in vivo* array studies (cohorts 1–3, Figure S1) were approved by the ethics board of University of Gothenburg and all participants provided written consent for participation. The quantification of CD4^+^ T-cell subtypes study (cohort 4, Figure S1), was approved by the ethics board of Linkoping University and all participants provided written consent for participation.

### Study subjects

We recruited patients with SAR and matched healthy controls of Swedish origin at The Queen Silvia Children's Hospital, Gothenburg (cohorts 1–3, Figure S1). We recruited patients with SAR and matched healthy controls of Swedish origin at Linkoping University Hospital, Linkoping (cohort 4, Figure S1). SAR was defined by a positive seasonal history and a positive skin prick test or by a positive ImmunoCap Rapid (Phadia, Uppsala, Sweden) to birch and/or grass pollen. Patients with perennial symptoms or asthma were not included. The healthy subjects did not have any history for SAR and had negative ImmunoCap Rapid tests. Supplemental Figure S1 provides an overview of the experiments performed on each cohort used in the research presented here. Severity of patient symptoms (itchiness of the eyes, block sinus, running nose) during season were self-assessed using a visual analogue scale (1–10). The score for each symptom was summed to give a single symptom severity score for each patient (Supplemental Table S1).

A detailed description of all methods employed in this study can be found in Supplemental Text S1.

## Supporting Information

Figure S1 Cohorts used in the course of this research. PBMCs from patients with SAR and age- and sex-matched healthy controls of Swedish origin were collected at The Queen Silvia Children's Hospital, Gothenburg & Linkoping University Hospital, Linkoping. SAR was defined by a positive seasonal history and a positive skin prick test or by a positive ImmunoCap Rapid (Phadia, Uppsala, Sweden) to birch and/or grass pollen. Patients with perennial symptoms or asthma were not included. The healthy subjects did not have any history for SAR and had negative ImmunoCap Rapid tests.
(TIF)

Figure S2 The DNA methylation profile of allergen-challenged CD4^+^ T-cells separates SAR patients from healthy controls. Principle components analysis of gene expression (*N_patients_* = 21, *N_controls_* = 21) and DNA methylation data (*N_patients_* = 12, *N_controls_* = 12) of CD4^+^ T-cells isolated after (**A**) diluent-challenge or (**B**) allergen-challenge of PBMCs from patients and healthy control subjects collected outside the pollen season. Clear separation is only observed by DNA methylation profile after allergen-challenge.
(TIF)

Figure S3 Functional analysis of *in vivo* CD4^+^ T-cell gene expression changes observed between SAR patients and healthy controls. (**A**) Gene Ontology enrichment analysis of genes gene miss-expressed (Mann-Whitney *U*-test; *P*<0.05, unadjusted for multiple testing) between patients and controls both during and outside the pollen season. (**B**) Boxplot showing expression levels of genes miss-expressed in patients versus controls. Genes are grouped by the direction of change of promoter methylation of each gene. No significant (*P*<0.05) difference was detected between the groups using a Mann-Whitney *U*-test.
(TIF)

Figure S4 Validation of DNA methylation changes between SAR patients and controls by pyrosequencing. (**A**) Distribution of observed methylation changes between SAR patients and healthy controls across all probes. Position of standard deviation (σ) is shown in red. (**B**) Volcano plot of median methylation differences of the 1,000 most significantly altered probes. (**C**) Percentage methylation values determined by pyrosequencing of 5 CpG loci in four patients and four controls are highly significantly correlated with those determined by 450k methylation array (Spearman's *rho* = 0.96, P<10^−6^, *N* = 37). Pyrosequencing of control (**D**) methylated (CD74 promoter CpG) and (**E**) unmethylated (GAPDH promoter CpG) CpGs. (**F–H**) Pyrosequencing of three CpG sites identified as altered significantly (in top 50 altered probes) between patients and controls by DNA methylation array. Pyrosequencing of CpG sites *CD74*, *RPP21* and *HLA-DMA* failed in sample 75P due to inefficient amplification, insufficient DNA was available to repeat these three assays.
(TIF)

Figure S5 Genic location and functional annotation of DNA methylation changes between SAR patients and controls. (**A**) Distribution of probes in various genic compartments across the entire 450K array (left panel) and of the 1,000 most significantly altered probes (right panel). (**B**) Bar graph of showing the enrichment of differentially methylated probes between patients and controls in enhancer elements. DHS, DNaseI hypersensitive site. Probes with significantly altered methylation between patients and controls. (**C**) Venn diagram showing significant overlap of differentially methylated enhancer probes between patients and controls identified both during and after the pollen season. (**D**) Enhancer probes show a strong trend towards loss of methylation in patients versus controls. (**E**) Gene Ontology enrichment analysis of genes containing the top 1,000 most significantly altered probes (left panel), and the top 100 most significantly altered probes (right panel).
(TIF)

Figure S6 Gating strategy employed for quantification of CD4^+^ T-cell subtypes. (**A**) Total CD4^+^ T cells were gated as CD4^+^SSClow population, (**B**) in which naïve CD4^+^ T cells (NT, CCR7^+^CD45RA^+^), CD4+ T central memory cells (TCM, CCR7^+^CD45RA^−^) and CD4^+^ T effector memory cells (TEM, CCR7^−^CD45RA^−^) were gated.
(TIF)

Table S1 Characteristics of subjects used in analysis of *in vivo* CD4+ T-cell DNA methylation by Infinium 450k array.
(DOCX)

Table S2 Pyrosequencing oligonucleotides.
(DOCX)

Text S1 Supplementary methods.
(DOCX)
