# Supplementary figures and images for: DNA Methylation Changes Separate Allergic Patients from Healthy Controls and May Reflect Altered CD4+ T-Cell Population Structure
Source: PLoS Genet. 2014 Jan 2;10(1):e1004059. doi: 10.1371/journal.pgen.1004059 (PMC3879208; doi:10.1371/journal.pgen.1004059)

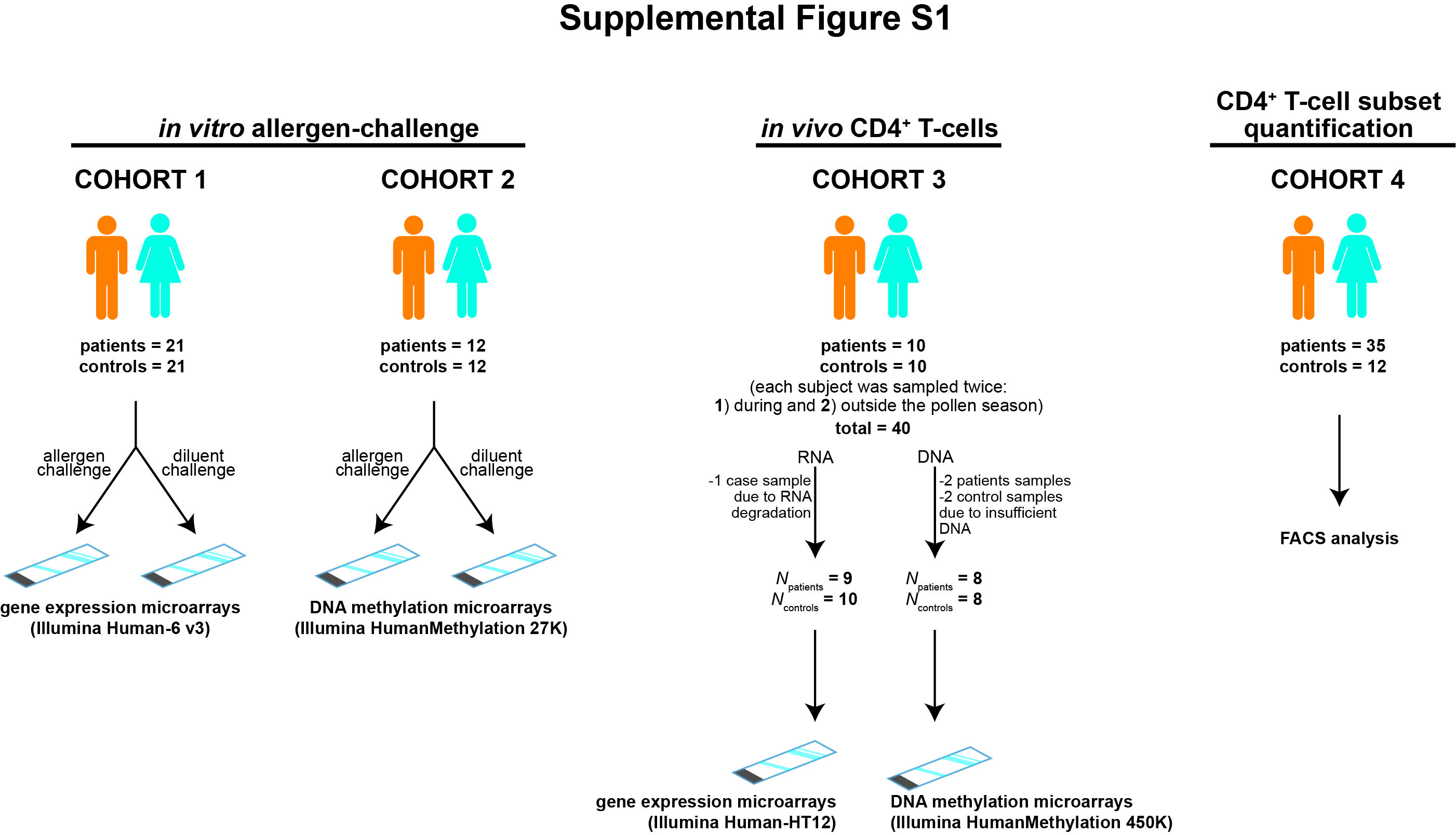

Supplement: Figure S1 — Cohorts used in the course of this research. PBMCs from patients with SAR and age- and sex-matched healthy controls of Swedish origin were collected at The Queen Silvia Children's Hospital, Gothenburg & Linkoping University Hospital, Linkoping. SAR was defined by a positive seasonal history and a positive skin prick test or by a positive ImmunoCap Rapid (Phadia, Uppsala, Sweden) to birch and/or grass pollen. Patients with perennial symptoms or asthma were not included. The healthy subjects did not have any history for SAR and had negative ImmunoCap Rapid tests. (TIF) [file pgen.1004059.s001.tif]

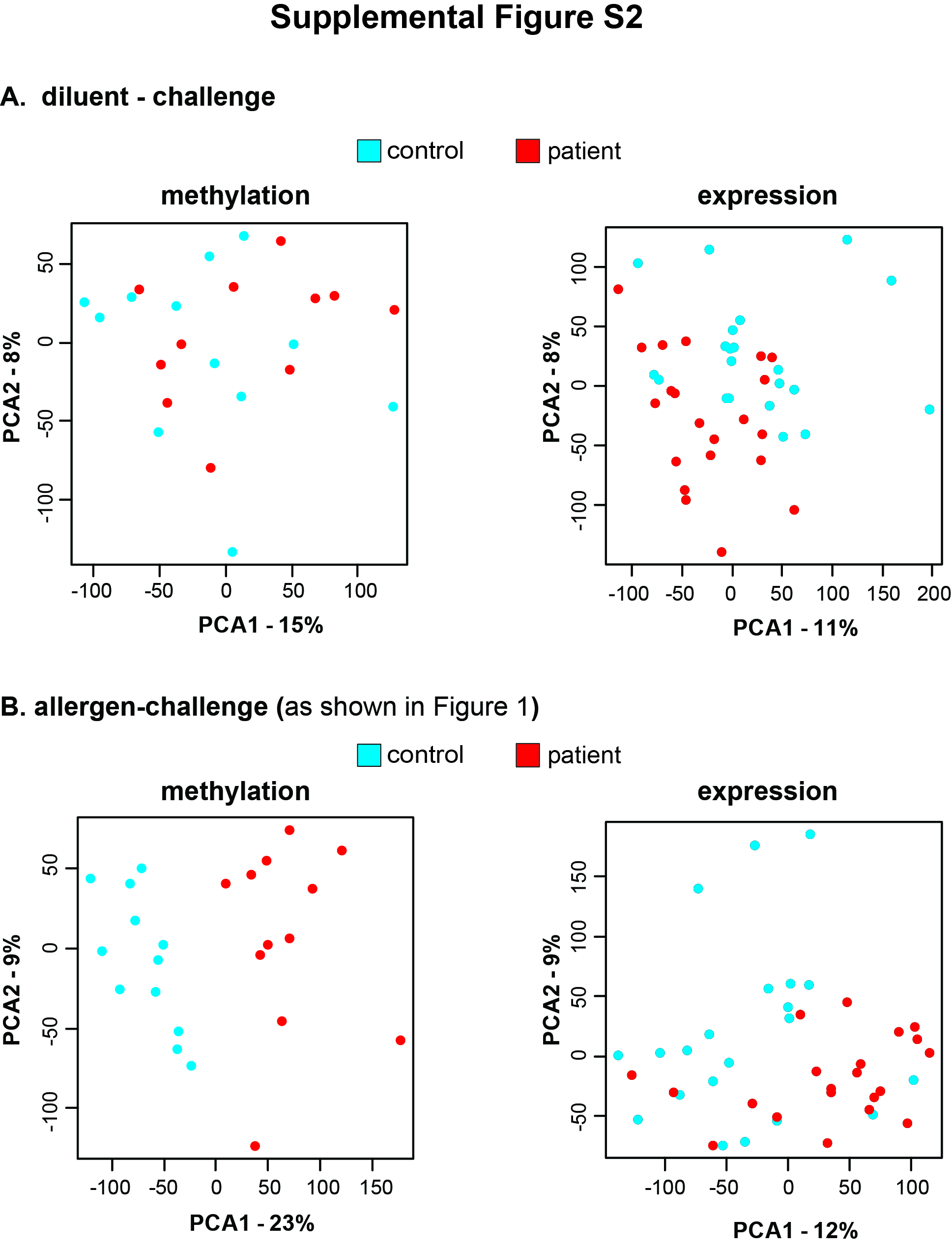

Supplement: Figure S2 — The DNA methylation profile of allergen-challenged CD4+ T-cells separates SAR patients from healthy controls. Principle components analysis of gene expression (Npatients = 21, Ncontrols = 21) and DNA methylation data (Npatients = 12, Ncontrols = 12) of CD4+ T-cells isolated after (A) diluent-challenge or (B) allergen-challenge of PBMCs from patients and healthy control subjects collected outside the pollen season. Clear separation is only observed by DNA methylation profile after allergen-challenge. (TIF) [file pgen.1004059.s002.tif]

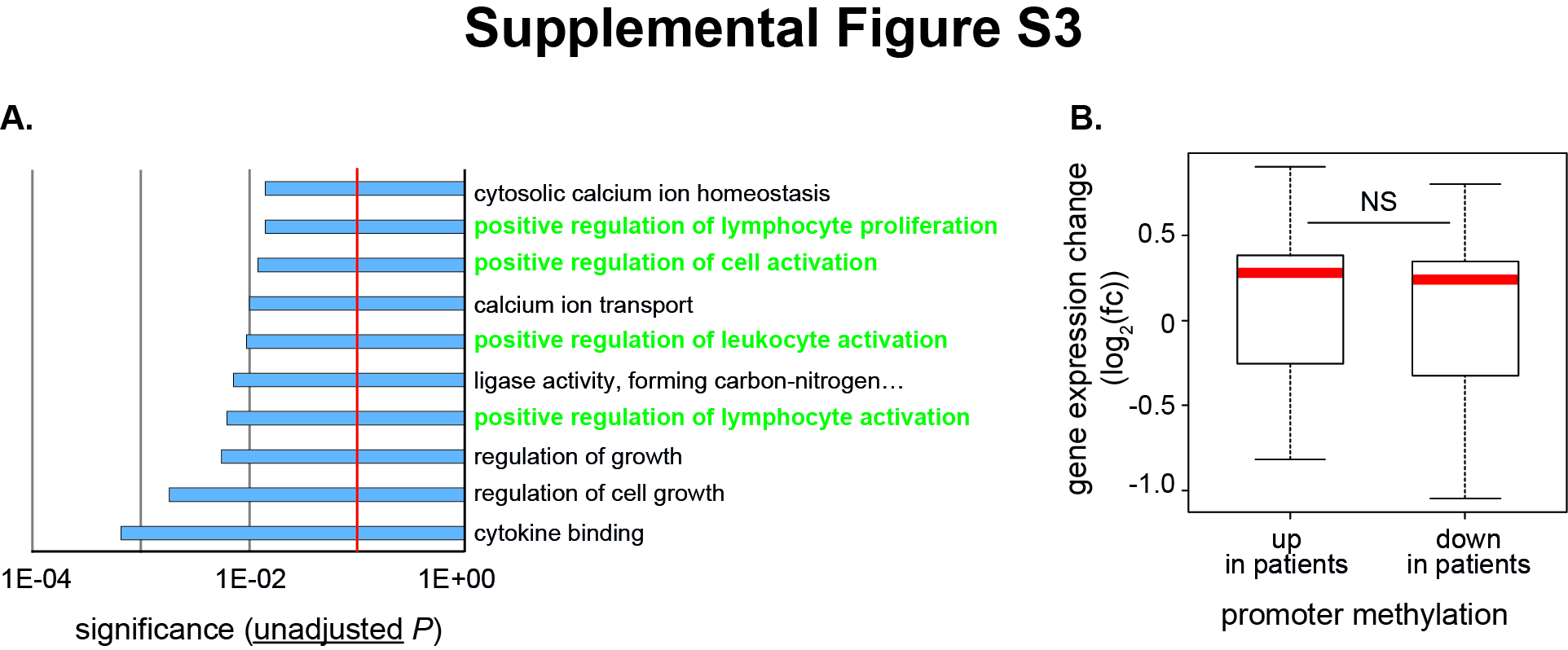

Supplement: Figure S3 — Functional analysis of in vivo CD4+ T-cell gene expression changes observed between SAR patients and healthy controls. (A) Gene Ontology enrichment analysis of genes gene miss-expressed (Mann-Whitney U-test; P<0.05, unadjusted for multiple testing) between patients and controls both during and outside the pollen season. (B) Boxplot showing expression levels of genes miss-expressed in patients versus controls. Genes are grouped by the direction of change of promoter methylation of each gene. No significant (P<0.05) difference was detected between the groups using a Mann-Whitney U-test. (TIF) [file pgen.1004059.s003.tif]

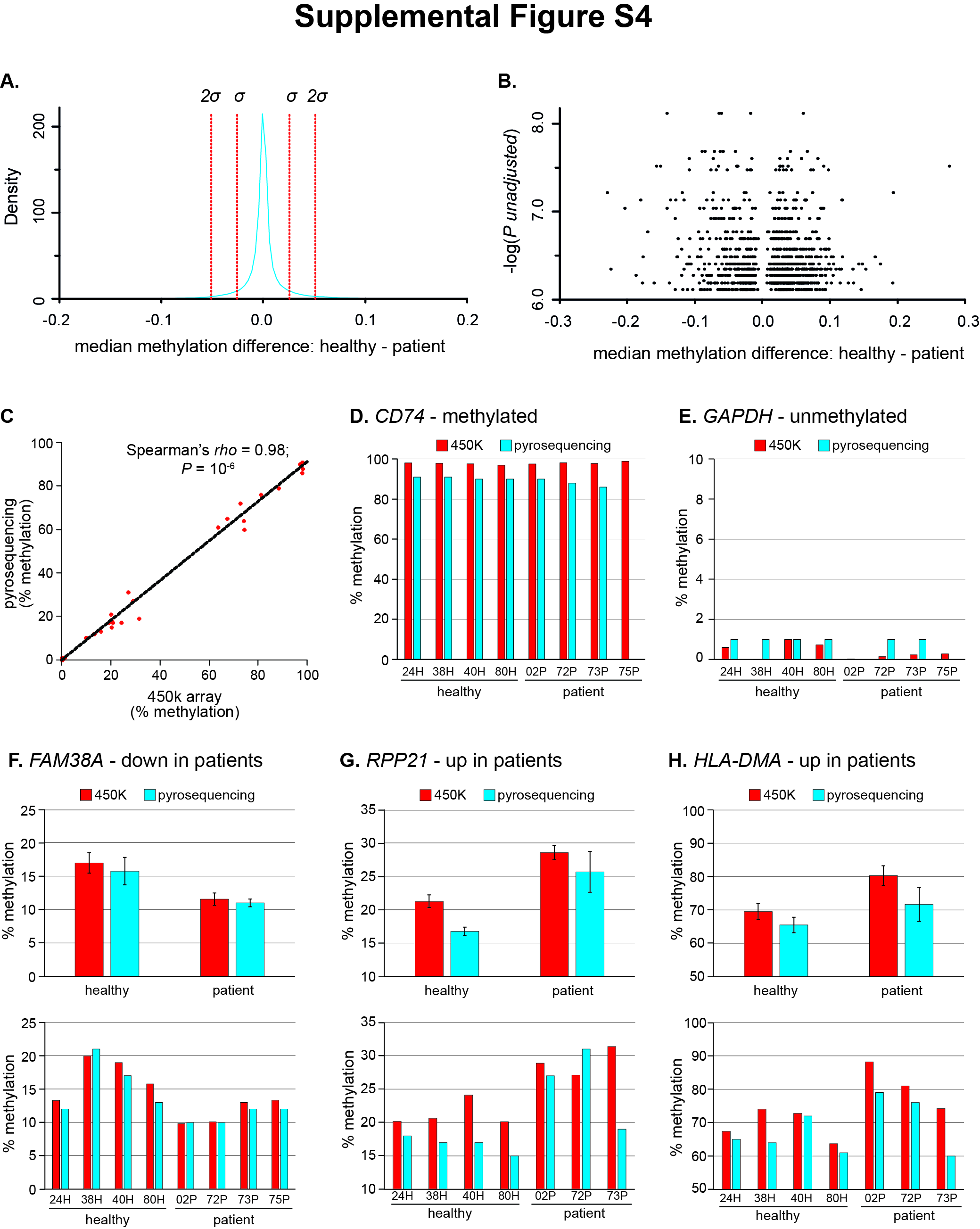

Supplement: Figure S4 — Validation of DNA methylation changes between SAR patients and controls by pyrosequencing. (A) Distribution of observed methylation changes between SAR patients and healthy controls across all probes. Position of standard deviation (σ) is shown in red. (B) Volcano plot of median methylation differences of the 1,000 most significantly altered probes. (C) Percentage methylation values determined by pyrosequencing of 5 CpG loci in four patients and four controls are highly significantly correlated with those determined by 450k methylation array (Spearman's rho = 0.96, P<10−6, N = 37). Pyrosequencing of control (D) methylated (CD74 promoter CpG) and (E) unmethylated (GAPDH promoter CpG) CpGs. (F–H) Pyrosequencing of three CpG sites identified as altered significantly (in top 50 altered probes) between patients and controls by DNA methylation array. Pyrosequencing of CpG sites CD74, RPP21 and HLA-DMA failed in sample 75P due to inefficient amplification, insufficient DNA was available to repeat these three assays. (TIF) [file pgen.1004059.s004.tif]

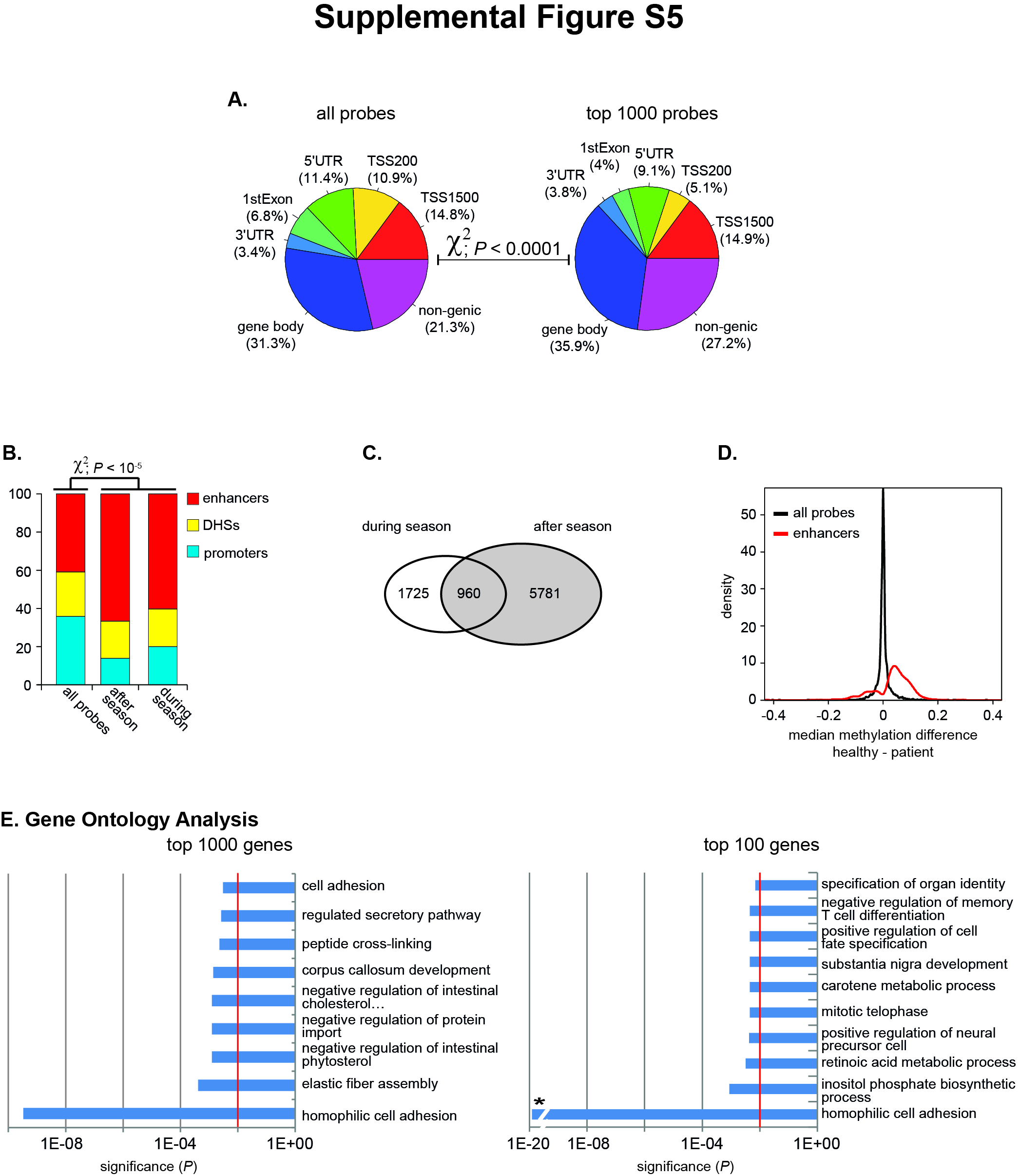

Supplement: Figure S5 — Genic location and functional annotation of DNA methylation changes between SAR patients and controls. (A) Distribution of probes in various genic compartments across the entire 450K array (left panel) and of the 1,000 most significantly altered probes (right panel). (B) Bar graph of showing the enrichment of differentially methylated probes between patients and controls in enhancer elements. DHS, DNaseI hypersensitive site. Probes with significantly altered methylation between patients and controls. (C) Venn diagram showing significant overlap of differentially methylated enhancer probes between patients and controls identified both during and after the pollen season. (D) Enhancer probes show a strong trend towards loss of methylation in patients versus controls. (E) Gene Ontology enrichment analysis of genes containing the top 1,000 most significantly altered probes (left panel), and the top 100 most significantly altered probes (right panel). (TIF) [file pgen.1004059.s005.tif]

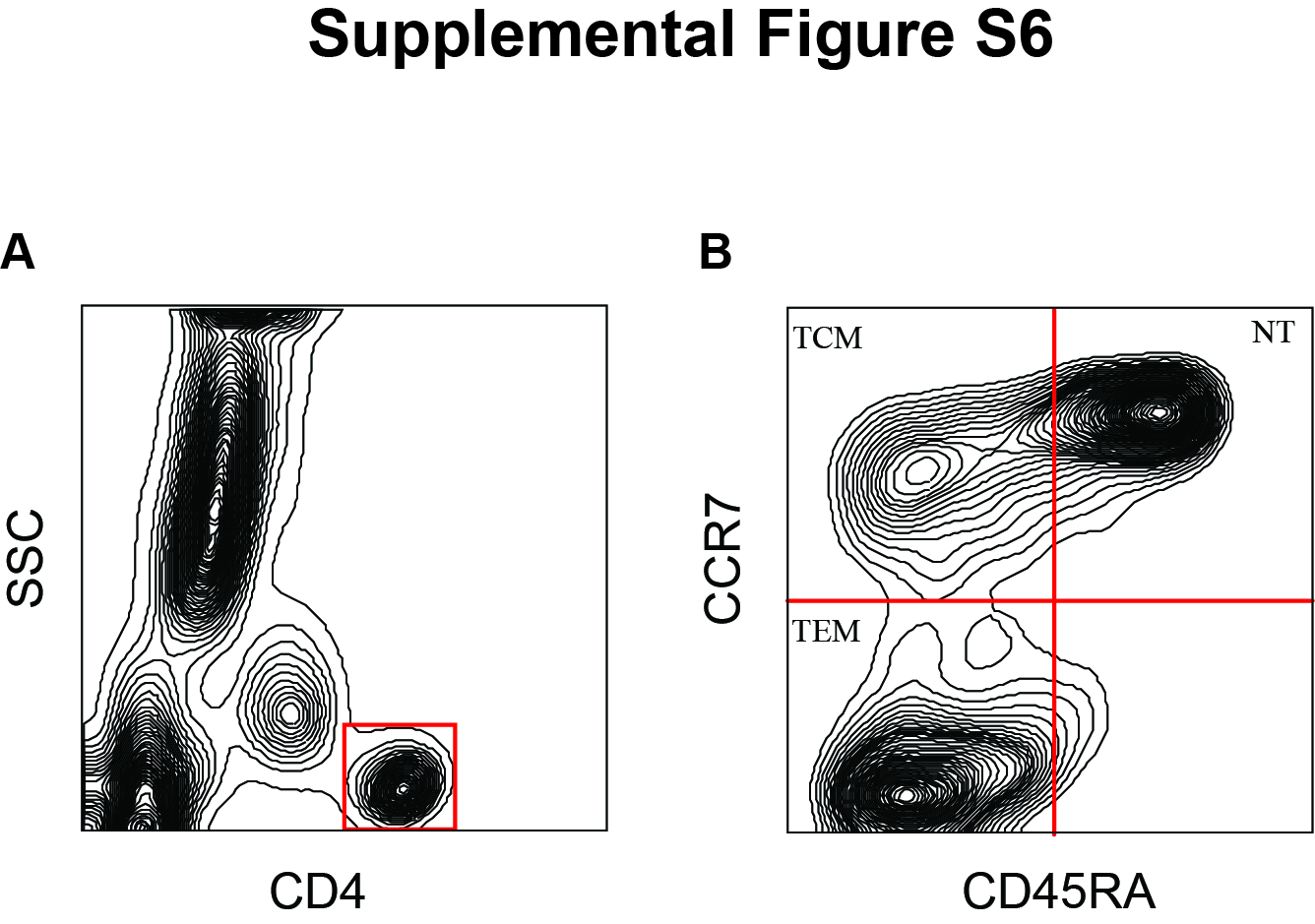

Supplement: Figure S6 — Gating strategy employed for quantification of CD4+ T-cell subtypes. (A) Total CD4+ T cells were gated as CD4+SSClow population, (B) in which naïve CD4+ T cells (NT, CCR7+CD45RA+), CD4+ T central memory cells (TCM, CCR7+CD45RA−) and CD4+ T effector memory cells (TEM, CCR7−CD45RA−) were gated. (TIF) [file pgen.1004059.s006.tif]
