# Supplementary material for: DNA Methylation Changes Separate Allergic Patients from Healthy Controls and May Reflect Altered CD4+ T-Cell Population Structure
Source: PLoS Genet. 2014 Jan 2;10(1):e1004059. doi: 10.1371/journal.pgen.1004059 (PMC3879208; doi:10.1371/journal.pgen.1004059)
Supplement: Table S1 — Characteristics of subjects used in analysis of in vivo CD4+ T-cell DNA methylation by Infinium 450k array. (DOCX) [file pgen.1004059.s007.docx]

| sample name | status | season^A^ | sex^B^ | array | runny nose^C^ | itch^D^ | block^E^ | symptom score^F^ | PCA1 | PCA2 |
| --- | --- | --- | --- | --- | --- | --- | --- | --- | --- | --- |
| 11H | control | OS | F | B | na | na | na | na | -211.7 | -54.3 |
| 24H | control | OS | F | B | na | na | na | na | -282.0 | 62.5 |
| 28H | control | OS | F | C | na | na | na | na | -215.7 | -63.4 |
| 32H | control | OS | M | A | na | na | na | na | 288.0 | -93.8 |
| 38H | control | OS | M | B | na | na | na | na | -231.2 | 253.4 |
| 40H | control | OS | M | C | na | na | na | na | -444.8 | 263.6 |
| 76H | control | OS | M | A | na | na | na | na | -152.7 | -377.9 |
| 80H | control | OS | M | A | na | na | na | na | -299.7 | -252.7 |
| 11H | control | DS | F | B | na | na | na | na | -182.0 | 20.7 |
| 24H | control | DS | F | B | na | na | na | na | -122.7 | 95.9 |
| 28H | control | DS | F | C | na | na | na | na | -231.7 | 3.5 |
| 32H | control | DS | M | A | na | na | na | na | 256.4 | -118.8 |
| 38H | control | DS | M | B | na | na | na | na | -260.4 | 274.3 |
| 40H | control | DS | M | C | na | na | na | na | -198.1 | 236.5 |
| 76H | control | DS | M | A | na | na | na | na | -155.9 | -323.1 |
| 80H | control | DS | M | A | na | na | na | na | -285.6 | -212.8 |
| 71P | patient | OS | M | A | na | na | na | na | 45.7 | -65.5 |
| 57P | patient | OS | F | A | na | na | na | na | 78.8 | -94.9 |
| 75P | patient | OS | F | B | na | na | na | na | -86.8 | 93.0 |
| 72P | patient | OS | F | B | na | na | na | na | 343.8 | 97.4 |
| 73P | patient | OS | M | B | na | na | na | na | 244.6 | -43.3 |
| 74P | patient | OS | M | C | na | na | na | na | 249.6 | 34.8 |
| 77P | patient | OS | M | C | na | na | na | na | 461.2 | 125.4 |
| 02P | patient | OS | F | A | na | na | na | na | 234.5 | -85.0 |
| 71P | patient | DS | M | A | 1 | 1 | 1 | 3 | -59.9 | -45.1 |
| 57P | patient | DS | F | A | 3 | 0 | 2 | 5 | 65.3 | -107.3 |
| 75P | patient | DS | F | B | 4 | 6 | 1 | 11 | -117.7 | 79.6 |
| 72P | patient | DS | F | B | 4 | 5 | 3 | 12 | 62.6 | 134.2 |
| 73P | patient | DS | M | B | 3 | 8 | 3 | 14 | 124.7 | -15.0 |
| 74P | patient | DS | M | C | 7 | 1 | 7 | 15 | 165.8 | 79.2 |
| 77P | patient | DS | M | C | 9 | 1 | 9 | 19 | 528.5 | 146.3 |
| 02P | patient | DS | F | A | 7  **^A^** OS = out of season; DS = during season  ^B^ F = female; M = male  **^F^** F = (C + D + E) | 9 | 8 | 24 | 389.2 | -47.5 |

**Supplemental Table 1.** Characteristics of subjects used in analysis of *in vivo* CD4+ T-cell DNA methylation by Infinium^®^ 450k array.
