# Supplementary material for: DNA Methylation Changes Separate Allergic Patients from Healthy Controls and May Reflect Altered CD4+ T-Cell Population Structure
Source: PLoS Genet. 2014 Jan 2;10(1):e1004059. doi: 10.1371/journal.pgen.1004059 (PMC3879208; doi:10.1371/journal.pgen.1004059)
Supplement: Table S2 — Pyrosequencing oligonucleotides. (DOCX) [file pgen.1004059.s008.docx]

|  | forward primer | reverse primer^A^ | sequencing primer | size (bp) |
| --- | --- | --- | --- | --- |
| *GAPDH* | GGAAAAAAAAAAGAGGGGAGAAAGTAGG | [Btn]CCCAACCCAAAATCTTAAAACCTAAACTA | GGGAGAAAGTAGGGT | 88 |
| *CD74* | TTATTTATAGATAGGGATATAGGGGTTTTT | [Btn]ATTCTCCTAAATCTAAACCACACAA | GGGTTTTTGTTTAGGATTATAT | 121 |
| *FAM38A* | GGGGATTTAGAGTAGTTTGTTTGAGGTTTT | [Btn]TTCCCCAAATTTCACCACCTTTCT | AGAGTAGTTTGTTTGAGGTTTTTA | 83 |
| *RPP21* | GGTTGTTTTTTAAAAGTTATTATTTATTGT | [Btn]ATTAAATTCCTAACCCCACTTACACAAACT | ATTATTTATTGTTTATATTGGAAGA | 104 |
| *HLA-DMA* | TAGGAGGGAGGAGAAGGTAG | [Btn]ACCACCTCCTAACAAAATACTAC | GTTTTGGGGGTTTATTTG | 92 |

**Table S2. Pyrosequencing oligonucleotides**

**^A^** Btn, biotinylated
