# Supplementary material for: DNA Methylation Changes Separate Allergic Patients from Healthy Controls and May Reflect Altered CD4+ T-Cell Population Structure
Source: PLoS Genet. 2014 Jan 2;10(1):e1004059. doi: 10.1371/journal.pgen.1004059 (PMC3879208; doi:10.1371/journal.pgen.1004059)
Supplement: Text S1 — Supplementary methods. (DOCX) [file pgen.1004059.s009.docx]

## *Supplemental methods*

## Allergen-challenge

Allergen challenge assays were performed as previously described [[1](#_ENREF_1),[2](#_ENREF_2)]. Briefly, PBMCs were prepared from fresh blood by means of centrifugation on Ficoll-Hypaque, washed, and stimulated with grass pollen extract (ALK-Abelló A/S; 100 μg/mL) at a density of 10^6^ cells/mL and in a total volume per well of 2 mL of cell culture medium or diluent (PBS). After 7 days of incubation at 37°C and 5% CO_2_, the supernatant was removed and CD4^+^ T-cells were enriched as described below.

**CD4^+^ T cell isolation**

Total CD4^+^ T cells were purified from PBMCs by MACS negative sorting using a Total CD4^+^ T cells Isolation Kit II (Miltenyi Biotec, Bergisch-Gladbach, Germany) for *in vivo* CD4^+^ T cells, or by positive sorting using anti-CD4-coated paramagnetic microbeads and a MACS system (Miltenyi Biotec GmbH, Bergisch Gladbach, Germany).

**Nucleic acid preparation and array hybridization**

RNA extraction was performed using a miRneasy Mini Kit (Qiagen). For each sample, 250 ng of total RNA was amplified and labeled using an Illumina TotalPrep 96 RNA Amplification Kit as per the manufacturer’s instructions. The quantity of the labeled cRNA was measured using a NanoDrop spectrophotometer, and the quality and size distribution of the labeled cRNA assessed using an Agilent 2100 Bioanalyzer. 750 ng of biotin labeled cRNA was hybridized to an Illumina HumanHT-12 v4 Expression BeadChip. The hybridization, staining, washing and scanning was performed as per the manufacturer’s instructions. Patient and control samples were randomized across microarray slides.

DNA was extracted using an Allprep DNA/RNA Mini Kit (Qiagen), and DNA quantity assessed by picogreen fluorometry. Subsequently, DNA was bisulfite converted, amplified, fragmented and hybridized to an Illumina HumanMethylation450 BeadChip and scanned according to the manufacturer’s instructions. Patient and control samples were randomized across microarray slides. All microarray processing was performed at the Microarray Core Facility, Rikshospitalet-Radiumhospitalet Medical Center, University of Oslo.

**Processing of gene expression microarray data**

The microarray platforms used in each experiment are shown in Supplemental Figure S1. Data from Illumina Human-6 v3 and Illumina Human-HT12 gene expression microarrays were analyzed using the R statistical programming language, using functions of the gene expression microarray analysis package, ‘lumi’ [[3](#_ENREF_3)]. Raw probe expression values were background subtracted, variance-stabilizing transformation was used to stabilize the expression variance [[4](#_ENREF_4),[5](#_ENREF_5)], and quantile normalization was used to normalize values across all arrays. This is a well-established and widely-used pipeline for the analysis of Illumina gene expression microarrays.

**Processing of DNA methylation microarray data**

The microarray platforms used in each experiment are shown in Supplemental Figure S1. Signal intensities were obtained by scanning of the 450K chips using an Illumina iScan. Signal intensities of methylated (M) and unmethylated beads (U) were converted to methylation scores (B);

$$\beta=\frac{U}{M +U +100}$$

As the 27K and 450K DNA methylation arrays contain different types of probe design, the data from each must be processed differently.

*Illumina 27K methylation array data*

Probes located on the X and Y chromosomes were removed to normalize for effects of sample gender. (2) Between array normalization was performed by quantile normalization. All remain probes were used downstream clustering and differential analyses.

*450K array data*

Unlike Illumina 450K methylation arrays, no single approach for the pre-processing and normalization of 450k methylation arrays is accepted. This complexity is primarily due to the fact that 450K methylation arrays are composed of two different types of probes; Type I and Type II probes. As **1**) Type I and Type II probes assay very different proportions of genomic sequence types (promoters, CGI islands, UTRs, etc) with different methylation profiles, and **2**) have been shown to have different dynamic ranges, these probe types must be normalized independently.

Our Illumina 450K methylation array analysis pipeline was as follows:

1. Probes located on the X and Y chromosomes were removed to normalize for effects of sample gender.
2. Probes containing common (>5% frequency) single nucleotide polymorphisms (SNPs) in proximity to the assayed cytosine were removed, to control for haplotype effects.
3. Inconsistent probes, probes which fail to detect a signal with high confidence (Mann-Whitney *U*-test; *P* < 0.01) in all samples, were removed; unlike gene expression arrays, all probes on a DNA methylation array should return a significantly detectable value.
4. We normalized for probe type using the published and validated peak-correction method [[6](#_ENREF_6)].
5. Finally, between array normalization was performed by quantile normalization of type I and type II probes separately across all arrays.

Our 450K processing pipeline, including separate quantile normalization of Type I and Type II probes has recently been shown to be among the most effective and accurate, in two independent studies of 450K processing [[7](#_ENREF_7),[8](#_ENREF_8)].

**Data analysis and statistics**

All data analysis was carried out using the R statistical programming language. All differential expression/methylation between groups was determined by the non-parametric Mann-Whitney *U*-test.

Unsupervised hierarchical clustering of both gene expression and DNA methylation data was performed on all probes remaining after the preprocessing steps outlined above; no further sub selection of probes was performed. Clustering was performed using the ‘hclust’ function. A distance matrix was computed for each data set using Euclidean distance, subsequently samples were clustered using Ward's minimum variance agglomeration method. The reproducibility and stability of the observed clusters was tested by consensus clustering whereby increasingly large subsets of the data are repeatedly re-sampled and re-clustered using the package ‘pvclust’.

Principle components analyses (PCA) of both gene expression and DNA methylation data was performed on all probes remaining after the preprocessing steps outlined above; no further sub selection of probes was performed. Principle components analyses was performed on the exact same datasets used for hierarchical clustering. The principle components were obtained using the ‘PCA’ function of the FactoMineR package in R using the default parameters.

**Pyrosequencing**

Pyrosequencing was carried out at The Genome Centre, University of London. 500 ng genomic DNA was bisulfite converted using EZ-96 DNA kit TM (Zymo research) specified by the manufacturer. The converted DNA was PCR amplified using primers for each set of genes; the primers were designed using PyroMark Assay design 2.0. Primer sequences are listed Supplemental Table S2. The pyrosequencing was performed according to a published protocol using PSQ 96 MA (Qiagen) to get a percent methylation at a single CpG site. The percent methylation at each CpG was calculated using PyroQ-CpG 1.0.9.

**Determination of CD4^+^ T cell subset proportions**

Blood samples were collected from 35 patients with SAR and 9 healthy controls during the pollen season. The median (range) age of patients was 25 (16-54) and 13 were women, while the median (range) age of healthy controls was 25 (20-29) and 4 were women. Red blood cells were excluded by a Red blood lysis kit from Miltenyi Biotec (Bergisch-Gladbach, Germany). Leukocytes were labeled with Pacific Blue™ anti-human CD4, PE anti-human CCR7, FITC anti-human CD45RO and APC anti-human CD45RA (Biolegend, San Diego, CA, USA). The flow cytometry was performed using FACSAria flow cytometer (BD Biosciences, San Diego, CA, USA) and the data was analyzed by FlowJo 7.6 (Treestar, Inc., San Carlos, CA). The strategy follows that employed in other published studies [[9](#_ENREF_9)].

***References***

1. Benson M, Langston MA, Adner M, Andersson B, Torinssson-Naluai Å, et al. (2006) A network-based analysis of the late-phase reaction of the skin. Journal of Allergy and Clinical Immunology 118: 220-225.

2. Wang H, Barrenas F, Bruhn S, Mobini R, Benson M (2009) Increased IFN-gamma activity in seasonal allergic rhinitis is decreased by corticosteroid treatment. J Allergy Clin Immunol 124: 1360-1362.

3. Du P, Kibbe WA, Lin SM (2008) lumi: a pipeline for processing Illumina microarray. Bioinformatics 24: 1547-1548.

4. Dunning MJ, Ritchie ME, Barbosa-Morais NL, Tavare S, Lynch AG (2008) Spike-in validation of an Illumina-specific variance-stabilizing transformation. BMC Res Notes 1: 18.

5. Lin SM, Du P, Huber W, Kibbe WA (2008) Model-based variance-stabilizing transformation for Illumina microarray data. Nucleic Acids Res 36: e11.

6. Dedeurwaerder S, Defrance M, Calonne E, Denis H, Sotiriou C, et al. (2011) Evaluation of the Infinium Methylation 450K technology. Epigenomics 3: 771-784.

7. Marabita F, Almgren M, Lindholm ME, Ruhrmann S, Fagerstrom-Billai F, et al. (2013) An evaluation of analysis pipelines for DNA methylation profiling using the Illumina HumanMethylation450 BeadChip platform. Epigenetics 8: 333-346.

8. Pidsley R, CC YW, Volta M, Lunnon K, Mill J, et al. (2013) A data-driven approach to preprocessing Illumina 450K methylation array data. BMC Genomics 14: 293.

9. McKinney EF, Lyons PA, Carr EJ, Hollis JL, Jayne DR, et al. (2010) A CD8+ T cell transcription signature predicts prognosis in autoimmune disease. Nat Med 16: 586-591, 581p following 591.
